# IgG4-related disease - focus on digestive system involvement

**DOI:** 10.3389/fimmu.2025.1584107

**Published:** 2025-06-18

**Authors:** Jakub Motor, Agata Gajewska, Krzysztof Cienkowski, Sara Langner, Łukasz Durko, Ewa Malecka-Wojciesko

**Affiliations:** ^1^ Clinical Department of General and Oncological Gastroenterology and Internal Medicine, Norbert Barlicki Memory University Teaching Hospital, Medical University of Lodz, Łódź, Poland; ^2^ Dr. Karol Jonscher City Medical Centre in Lodz, Łódź, Poland; ^3^ Central Teaching Hospital, Medical University of Lodz, Łódź, Poland; ^4^ Department of Digestive Tract Diseases, Medical University of Lodz, Łódź, Poland

**Keywords:** IgG4-RD, IgG4-related disease, autoimmune disease, autoimmune pancreatitis, IgG4-related cholangitis, digestive system, steroid therapy

## Abstract

IgG4-related disease (IgG4-RD) is a systemic fibroinflammatory condition characterized by the infiltration of IgG4-positive plasma cells in affected tissues, leading to fibrosis and progressive organ dysfunction. This review explores the epidemiology, pathogenesis, and organ manifestations of IgG4-RD, with a focus on autoimmune pancreatitis and sclerosing cholangitis as the main clinical presentations. It may cause exocrine and endocrine pancreatic insufficiency and chronic hepatobiliary failure. Main diagnostic challenges include differentiation from malignancies and other inflammatory conditions. Diagnosis of IgG4-RD involves combination of clinical symptoms, typical imaging findings, elevated serum IgG4 levels, and histopathological evidence of IgG4-positive plasma cell infiltration. Advances in clinical understanding of the disease, histopathological and serological markers, imaging techniques, have enhanced early detection. Current treatment strategies prioritize steroids therapy for induction of remission, while steroid-sparing agents, including disease-modifying antirheumatic drugs and rituximab play the pivotal roles in managing its relapses or steroid-resistant disease. Biologic therapies are also promising therapeutic avenues. In addition, multidisciplinary approach optimizes the diagnosis, treatment, and long-term outcomes in this complex disease.

## 1 Introduction

Immunoglobulin G4-related disease (IgG4-RD) is the fibroinflammatory disorder marked by the presence of IgG4-positive plasma cells infiltrating tissues, which results in damage to affected organs. Patients typically exhibit elevated levels of serum IgG4. The disease is characterized by distinctive histopathological features, including storiform fibrosis and obliterative phlebitis (1). This condition can involve any anatomical region, with the pancreas, lacrimal glands, salivary glands, and retroperitoneum being the most frequently affected areas. Around 40% of patients present with the single organ disease, although cases involving six organs are not rare and this number may increase with time. In IgG4-RD, the pancreas is commonly the most affected organ, with studies in the United States indicating that up to 70% of patients diagnosed with IgG4-RD show signs of pancreatic involvement, manifested as autoimmune pancreatitis (AIP) (2). Involvement of the skin, prostate, or peripheral nerves is extremely rare (3). Data regarding the frequency of involvement of specific organs will vary across different studies, depending on the continent where the research was conducted and due to the relatively small patient groups, considering that the described disease entity is rare. IgG4-related disease is the slowly progressing disease, may remain asymptomatic for years and may can cause organ failure even before the diagnosis.

## 2 Epidemiology

The increased detection of IgG4-related disease is due to the advancements in diagnostic methods and the growing awareness among clinicians due to the enormous increase of knowledge in this area within the last decade. The study conducted in the United States by Wallace ZS et al. showed that the incidence of IgG4-RD increased from 0.78 to 1.39 per 100,000 person-years between 2015 and 2019 (2). The disease affects men about 1.6 times more often than women in head and neck regions, while in other areas, such as internal organs, men are impacted up to four times as often as women (4). IgG4-RD typically affects middle aged and older people - typically those in their 50s to 70s (5).

## 3 Etiology and pathogenesis

The role of genetic factors predisposing individuals to the development of IgG4-RD has been emphasized. Terao et al. identified several genetic loci linked to IgG4-RD in the Japanese population. Specifically, the authors highlighted associations with the HLA-DRB1*04:05 allele and other immune disease-related genes. These findings suggest that these genetic variations may contribute to the susceptibility of IgG4- RD (6).

**Figure 1:**
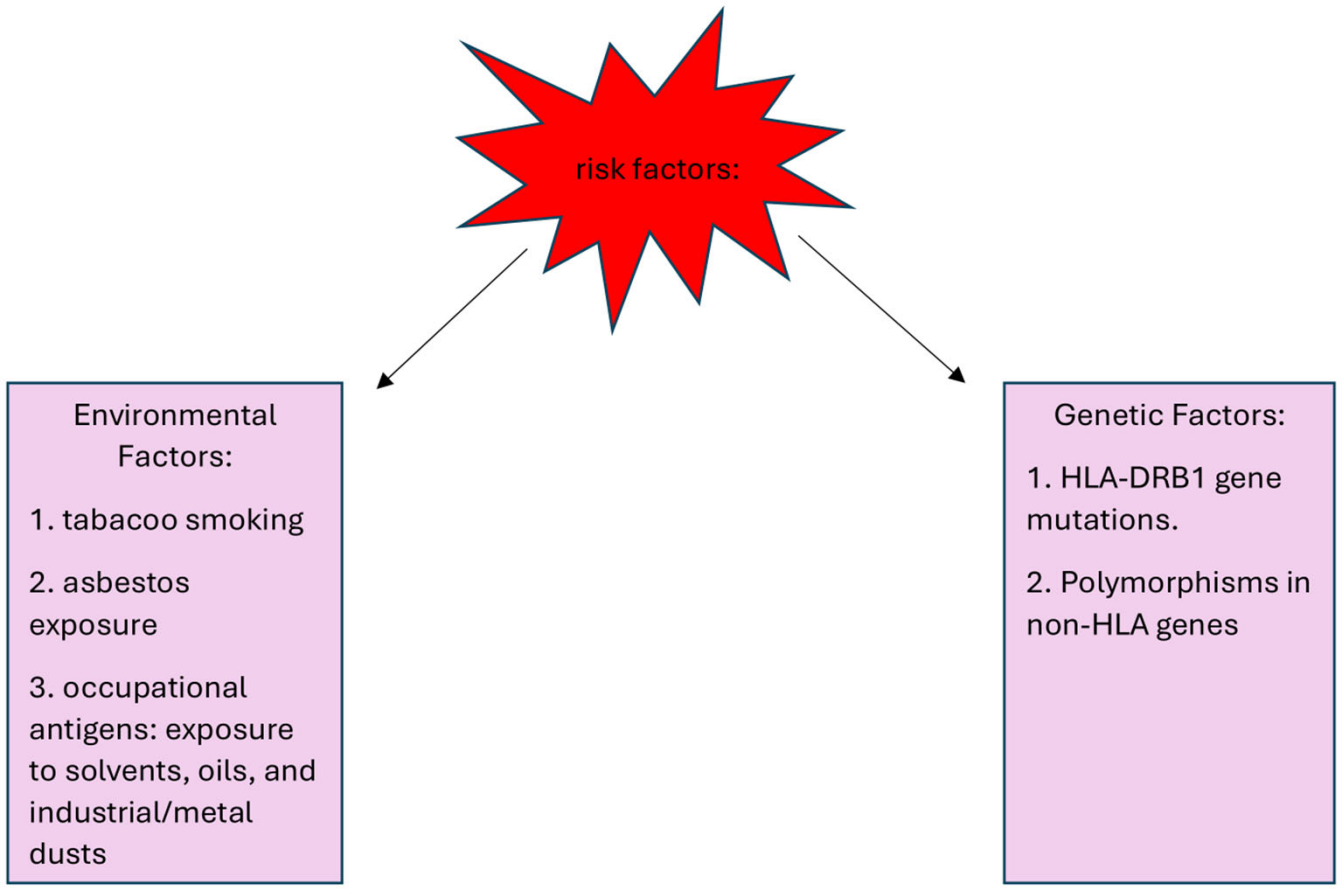
Risk factors for the development of IgG4-RD (7, 8).

Environmental factors include asbestos, oils, solvents and industrial and metal dust, which might trigger immune responses that predispose to IgG4-RD. Additionally, other risk factors, such as tobacco smoke, pose a risk to the disease risk in the broader population. Risk factors for the development of IgG4-RD are shown in **Figure 1**. de Buy Wenniger et al. carried out a structured questionnaire thoroughly assessing the job history of predominantly retired IgG4-RD patients. In the Amsterdam cohort, which included 25 patients with IRC (IgG4‐related cholangitis) and/or AIP (autoimmune pancreatitis), 88% had worked in blue-collar professions for at least one year, often spanning their entire careers. Among the occupations reported, chronic exposure to solvents, industrial and metal dust, pigments, and oils used in the automotive industry were identified as the most common occupational hazards. In contrast, among a control group of 21 patients with primary sclerosing cholangitis (PSC) only 14% had a history of blue-collar work. A similar assessment was conducted in the Oxford cohort, consisting of 44 patients with established IgG4-RD. Here, 61% had worked in blue-collar professions, and 52% reported chronic exposure to solvents, industrial dust, pesticides, or industrial oils and polymers. In comparison, among a control group of 27 PSC patients from Oxford, only 7% reported any exposure to these substances, often described as incidental (7).

The pathogenesis of IgG4-RD involves a cascade of immune cell activations and interactions, driving chronic inflammation and fibrosis in affected tissues. Characteristic of IgG4-RD is that, after initial immune activation, T follicular helper (Tfh) cells and regulatory T cells (Tregs) become activated, promoting the production of IgG4 antibodies. Tregs, which typically help control and suppress excessive immune responses, may function abnormally in IgG4-RD, potentially failing to regulate inflammation effectively (9). Next, B cells, once activated by Tfh cells, differentiate into plasma cells that produce high levels of IgG4 antibodies (10). Chronic activation of B and T cells sustains inflammation in affected tissues, leading to fibrosis as immune cells and cytokines stimulate fibroblasts. This excessive fibrotic tissue disrupts organ structure and function, causing visible lesions and potential long-term impairment. A hallmark of this process is storiform fibrosis, a whorled, cartwheel-like collagen pattern often seen in biopsies. Over time, normal tissue is replaced with fibrous tissue, progressively damaging affected organs (11, 12). It is increasingly recognized that the etiology and immunological characteristics of the disease onset vary depending on the clinical phenotype. Akiyama et al. used two classification systems for patients. In the first, they divided patients based on the affected organs, and in the second, based on allergic and malignant phenotypes. Each phenotype demonstrates different dominant subsets of immune cells involved in their pathogenesis. Organ-based classification includes:

1. Pancreato-hepatobiliary disease (31%)
2. Retroperitoneal fibrosis with or without aortitis (24% cases): characterized by a predominant presence of CX3CR1+ cytotoxic T lymphocytes (CTLs) in the affected tissues, which play a central role in the development of fibrosis in the affected areas.
3. Head and neck-limited disease (24%)
4. Classic Mikulicz’s disease with systemic involvement (22%): marked by an increase in follicular helper T cells (Tfh2), involved in promoting B cell activation and immunoglobulin production, contributing to the systemic manifestations of the disease, such as glandular enlargement.

Classification based on allergic and malignant phenotypes includes:

1. Malignant phenotype:
  characterized by an increased presence of CXCR5+ CD2 double-positive T cells. This subgroup may involve a more complex immune dysfunction, involving both inflammatory processes and associated with an increased risk of complicating malignancies
2. Allergic phenotype:

characterized by the dominance of Tfh2 cells, which are involved in promoting allergic responses. It is often associated with more localized involvement and is frequently linked to atopic conditions, such as asthma or allergic rhinitis (13).

## 4 Symptoms and different organs diagnostics

The diagnostic criteria for IgG4-related disease in most organs are based on three key factors. Clinical and radiological features, which involve the combination of diagnostic criteria specific to affected organs and serological diagnosis, with serum IgG4 levels > 135 mg/dl. Histopathologic disease features include lymphocyte and plasma cell infiltration with fibrosis, particularly storiform fibrosis or obliterative phlebitis. Immunohistochemistry staining reveals the ratio of IgG4-positive plasma cells to IgG-positive cells > 40%, and more than 10 IgG4-positive plasma cells per high-powered field (17).

In 2010, the International Association of Pancreatology in Japan classified autoimmune pancreatitis into two types, AIP 1 and AIP 2. This classification was based on five key features: imaging of the pancreatic parenchyma and duct, serology, involvement of other organs, pancreatic histology, and an optional criterion—response to steroid therapy. However, in some cases, distinguishing between the subtypes may not be possible, leading to a diagnosis of AIP-not otherwise specified (18).

AIP 1 is the most prevalent form of IgG4-RD in the gastrointestinal tract. A nationwide epidemiological survey conducted in Japan in 2016 reported an incidence of this disease as 3.1 per 100,000 individuals and a prevalence of 10.1 per 100,000 individuals (19). AIP-1 is more common in older adults, with a male-to-female ratio of 3:1 and a mean age of 65 years. It predominantly affects populations in Asia, with lower prevalence in Europe and the United States. In contrast, AIP-2 has a balanced male-to-female ratio of 1:1, with a younger mean age of 40 years. AIP-2 is more frequently observed in Europe and the United States, whereas its occurrence in Asia is less common (20).

The symptoms of AIP 1, described as lymphoplasmatic sclerosing pancreatitis, are generally nonspecific. The most typical are pancreatic pain or obstructive jaundice. Pancreatic pain is typically described as deep and dull, located in the upper abdomen, often radiating to the back, and can be exacerbated by eating, particularly fatty foods. It results from inflammation, swelling, or obstruction of the pancreatic ducts. Obstructive jaundice, weight loss, general fatigue, attacks of acute pancreatitis, signs of diabetes mellitus, and/or symptoms related to extra-pancreatic lesions may occur. In addition, abnormalities in liver tests due to cholestasis resulting from pancreatic swelling are possible. The most common conditions linked to AIP 1 are sclerosing cholangitis, dacryosialoadenitis, retroperitoneal fibrosis, and hydronephrosis. Fever is rare and may point out to the different diagnosis (21).

**Figure 2:**
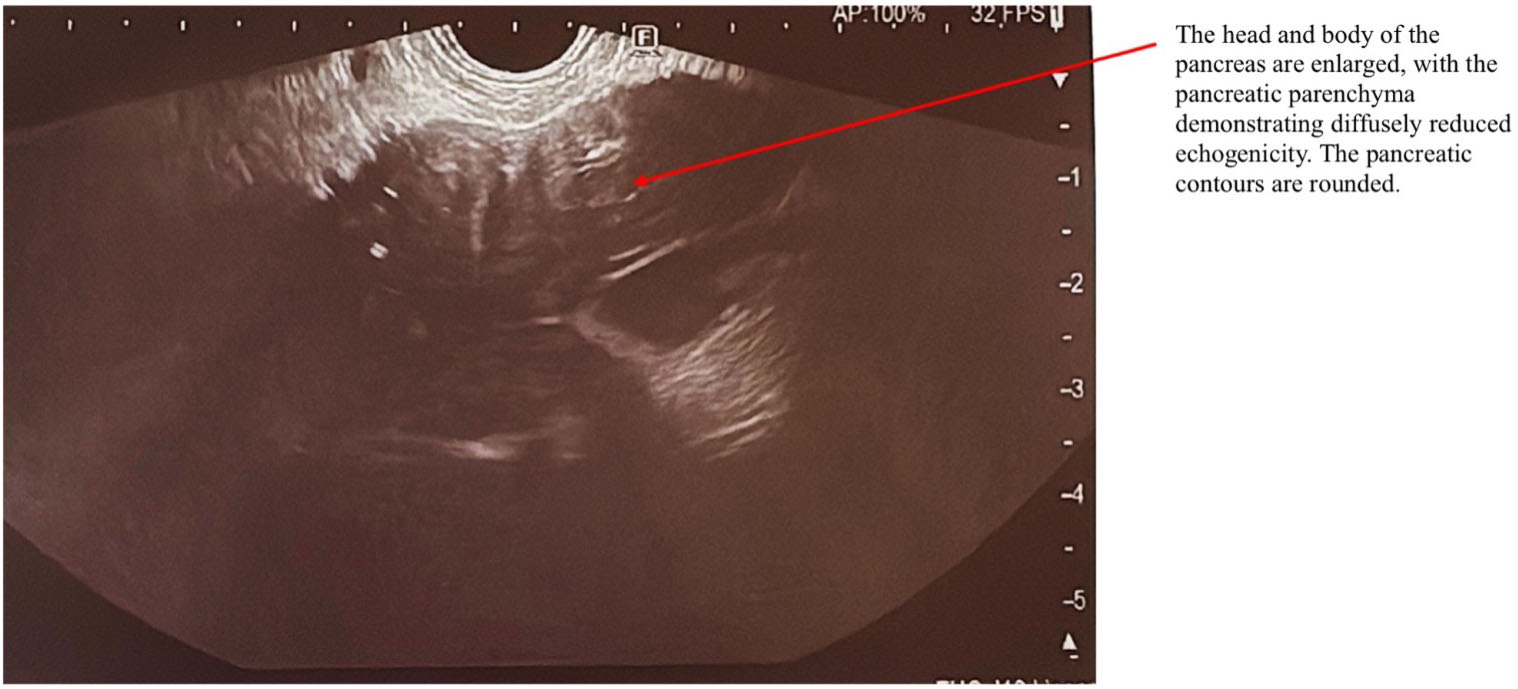
EUS examination of a patient with AIP. The arrow indicates the pancreas, which exhibits a characteristic “sausage-shaped” appearance. The head and body of the pancreas are enlarged, with diffusely reduced echogenicity of the pancreatic parenchyma. The pancreatic contours are rounded.

**Figure 3:**
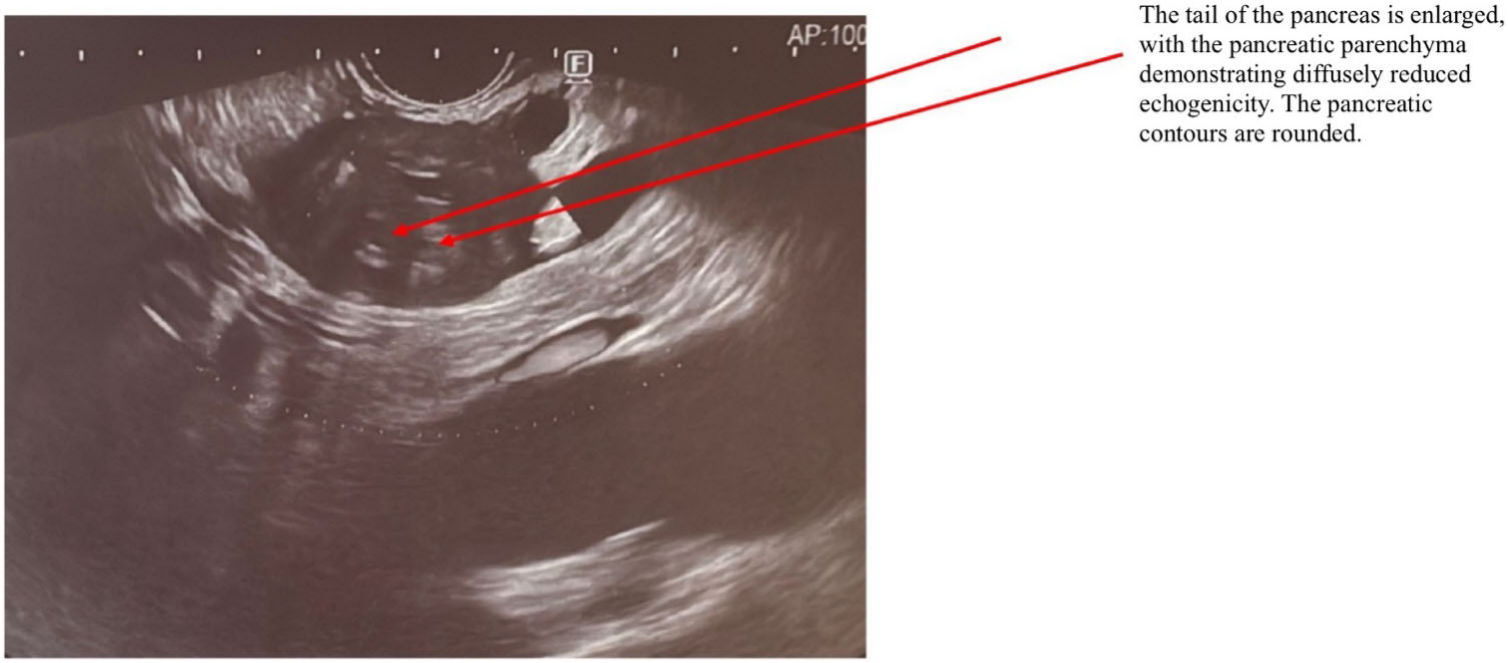
EUS examination of a patient with AIP. The arrow indicates the pancreas, which exhibits a characteristic “sausage-shaped” appearance. The tail of the pancreas is enlarged, with diffusely reduced echogenicity of the pancreatic parenchyma. The pancreatic contours are rounded.

**Figure 4:**
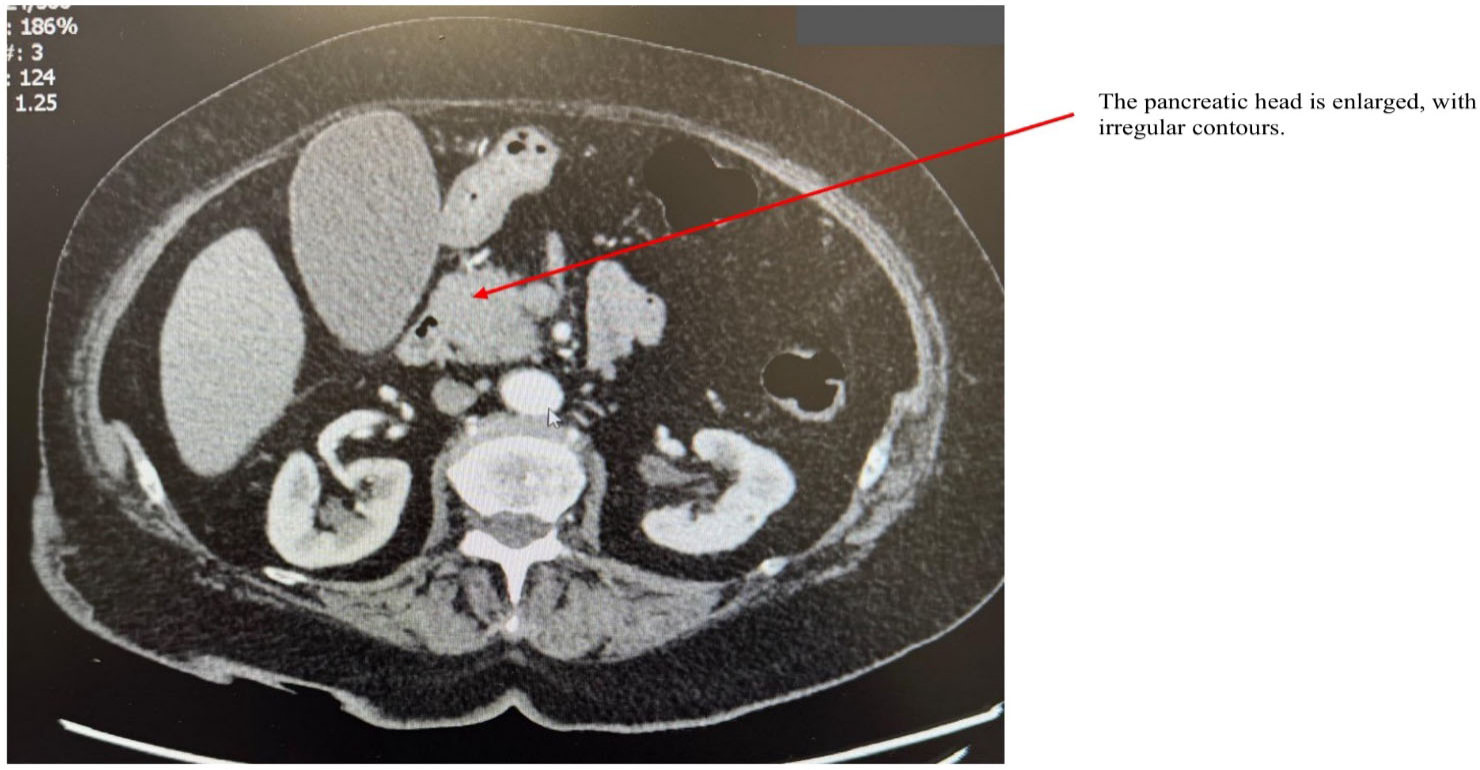
CT scan of a patient with AIP. The arrow points to an enlarged pancreatic head with irregular contours.

AIP1 is characterized radiologically by distinctive imaging features such as diffuse, segmental, or focal pancreatic enlargement, giving the pancreas a characteristic “sausage-like” appearance shown in **Figures 2**–**4** (22). Additional imaging findings include a capsule-like rim surrounding the pancreas, appearing as a low-attenuation area on CT or a hypointense ring on T1-weighted MRI, a hallmark of IgG4-RD (23). Magnetic resonance cholangiopancreatography (MRCP) may further distinguish AIP from pancreatic cancer or other diseases, demonstrating narrowing of the pancreatic duct without significant dilatation (23, 24).

AIP 2 is known as idiopathic duct-centric pancreatitis. This form is characterized by granulocytic epithelial lesions with neutrophil infiltration, which frequently leads to destruction and obliteration of the pancreatic duct (18, 25). Patients with type 2 AIP display similar pancreatic imaging to those with type 1 AIP but differ in clinicopathological features. The patients have abdominal pain and acute pancreatitis attacks. They generally have normal serum IgG4 levels, minimal or no IgG4-positive plasma cell infiltration, no serum IgG4 autoantibodies, and rarely have other organ involvement except for accompanying inflammatory bowel disease (IBD), seen in around 30% patients. For this reason, histopathological evaluation of the pancreas remains the important method for this type of AIP diagnosis (18).

The main EUS findings in AIP may be divided into diffuse and focal. In the diffuse AIP diffuse pancreatic enlargement with echo-poor echo texture, hyperechoic foci/stands or lobularity, loss of connection to the splenic vein, hyperechoic MPD walls thickening and peripancreatic hypoechoic margin; stones and cysts similar to those described in chronic alcoholic pancreatitis may occur in the late stages of AIP. In mass-forming AIP, EUS features include focal hypoechoic mass, absence of parenchymal heterogeneity, eventually PD dilation, and vessel involvement (26, 27).

Clinically, AIP and pancreatic cancer share many similarities, including a higher prevalence in elderly males, painless jaundice as a common initial symptom, new-onset diabetes mellitus, and elevated serum tumor markers. However, certain findings favor AIP over pancreatic cancer. These include fluctuating obstructive jaundice, elevated serum IgG4 levels, and diffuse pancreatic enlargement. Imaging features such as delayed enhancement of the pancreas and a capsule-like rim on dynamic CT, as well as irregular narrowing of the main pancreatic duct on ERCP, also support an AIP diagnosis. Additionally, the presence of other organ involvement, such as bilateral salivary gland swelling, retroperitoneal fibrosis, or hilar/intrahepatic sclerosing cholangitis, suggests AIP (28). Contrast-enhanced EUS (CH-EUS) may help distinguish AIP from pancreatic cancer (PDAC) showing hypervascularization in the first pathology and hypo vascularization in the latter. CH-EUS typically reveals focal or diffuse iso-enhancement in AIP and hypo-enhancement in PDAC. Additionally, pancreatic elastography-assisted EUS identifies uniform stiffness throughout the pancreas in AIP, distinguishing it from localized stiffness in PDAC. The particular role of endoscopic ultrasound (EUS) is due to its ability to biopsy the affected pancreatic parenchyma and provide the definite AIP diagnosis. EUS fine-needle core biopsy (FNB) is more accurate than fine-needle aspiration (FNA). Nevertheless the tissue sampling techniques for diagnosis of AIP remain unsatisfactory (29).

Histological findings are crucial for the diagnosis of IgG4-RD. Tissue samples for histological examination can be obtained during a biopsy when imaging studies such as CT or MRI reveal focal changes, and there is suspicion of a tumor. In such cases, the clinician decides to perform a biopsy. Histologically, AIP 1 is characterized by extensive infiltration of lymphocytes and IgG4-expressing plasma cells, storiform fibrosis, and obliterative phlebitis (30). Patients often exhibit elevated serum levels of IgG, and IgG4, as well as detectable autoantibodies, including anti-insulin antibodies, as well as other autoantibodies such as anti-amyloid A and anti-LKM-1 antibodies. Elevated serum IgG4 levels correlate with the severity of AIP symptoms, as higher IgG4 levels are associated with more frequent jaundice and pronounced pancreatic enlargement (31, 32). The vascular lesions characteristic of type 1 AIP, specifically obliterative phlebitis lead to the obliteration of venous structures, Parameters like neutrophilic abscesses and granulomas are typically absent in type 1 AIP (33).

According to the International Consensus Diagnostic Criteria based on nationwide epidemiological survey in Japan patients with sIgG4 levels ≥135 mg/dl at the diagnosis were classified as sIgG4-positive AIP 1, and those with sIgG4 levels <135 mg/dl were as sIgG4-negative AIP 1. Patients with sIgG4-positive and

sIgG4-negative type 1 AIP present with different clinicopathological features which suggests heterogeneity of patients with type 1 AIP. Low serum IgG4 levels could indicate low disease activity in type 1 AIP (34). The response to glucocorticoid therapy is also included in the diagnostic criteria.

The two defining features of AIP 2 are periductal lymphoplasmacytic infiltration and the presence of neutrophils within the ducts, known as granulocytic epithelial lesions - these neutrophils can also be found in the acinar epithelium, providing a helpful indication. The disease is also characterized by significant pancreatic atrophy and fibrosis, both in the interlobular septa and within the lobules. However, unlike AIP 1, there is a much less pronounced lymphoplasmacytic infiltration, especially outside the areas adjacent to the ducts. Additionally, the fibrosis lacks the typical storiform pattern seen in type-1 AIP. Obliterative phlebitis is absent. IgG4+ plasma cells are not present in AIP 2 (35).

Although AIP and PDAC are distinct conditions, it is worth noting that studies have shown an incidence of concomitant pancreatic tumors (both benign and malignant) in up to 7% of patients with AIP (36). It is crucial to note that IgG4+ plasma cells can be found in various connective tissue and neoplastic conditions, including PDAC. Notably, certain pancreatic adenocarcinomas exhibit a dense peritumoral infiltrate of IgG4+ plasma cells, and accidental sampling from this area might lead to a misdiagnosis of IgG4-related disease. This could delay the identification of cancer, potentially narrowing the already limited window for surgical resection (37).

Pancreatic lymphoma should be also considered in differential diagnosis, particularly when AIP presents with a mass. Pancreatic lymphoma is rare and often associated with systemic “B symptoms” such as fever, night sweats, and weight loss. Imaging may reveal homogenous, pancreatic and hypovascular mass. Tissue sampling can be performed using FNA, preferably under CT or EUS guidance. The most common histological type of primary pancreatic lymphoma (PPL) is diffuse large B-cell lymphoma (DLBCL), accounting for 80% of cases, though other histologic types may rarely occur. DLBCL is a high-grade, aggressive lymphoma characterized by large, atypical B cells diffusely infiltrating the tissue. Confirmation is achieved through immunohistochemical staining, demonstrating CD20-positive markers, which are indicative of B-cell lineage and are characteristic of DLBCL, a subtype of non-Hodgkin lymphoma (NHL) (38).

UEG guidelines recommend that AIP treatment should be considered for all symptomatic patients, such as those experiencing pancreatic pain, and may require urgent intervention, such as biliary stenting in cases of obstructive jaundice, severe pancreatic involvement, or other organ involvement. For asymptomatic patients, treatment may be warranted in specific situations, including:

1. The presence of a persistent pancreatic mass suggesting PDAC
2. Ongoing abnormalities in liver tests (cholestasis) associated with IRC
3. Subclinical conditions that could progress to severe or irreversible organ damage.

Currently, there is no substantial evidence to support treating asymptomatic AIP patients solely to prevent the onset of exocrine or endocrine insufficiencies (39).

AIP also demonstrates a strong response to steroid therapy, as evidenced by the rapid resolution of clinical symptoms such as abdominal pain and jaundice, reduction in pancreatic swelling, and improvement in biochemical markers, including CRP, serum IgG4 levels, and bilirubin levels in patients with jaundice (25, 40). It is important to emphasize that AIP is the only type of pancreatitis that responds to glucocorticoid treatment so biliary stenting is not always necessary for managing obstructive jaundice. Shimosegawa et al. conducted a study on 96 patients with type 1 AIP, showing that treatment with an initial dose of 0.6 mg/kg of prednisolone alone has been shown to resolve jaundice completely within 15 days, accompanied by a rapid normalization of liver function tests (18). Although glucocorticoids are effective, around one-third of patients experience a relapse of the disease during dose tapering, necessitating re-induction therapy. This typically includes increasing the steroid dose up to 1 mg/kg per day, followed by a gradual tapering to a minimum dose of 5 mg per day (41). Relapses can occur in pancreas or in other organs previously unaffected (42).

Moreover, the UEG guidelines for the diagnosis and treatment of AIP emphasize the importance of screening for deficiencies and supplementing of fat-soluble vitamins (A, D, E, and K), zinc, calcium, and magnesium (43). In addition, patients should undergo regular screening for pancreatic endocrine and exocrine insufficiency. Complications arising from pancreatic exocrine and endocrine insufficiency, such as malnutrition, diabetes mellitus, malabsorption, and osteopenia/osteoporosis, should be managed following British Society of Gastroenterology or United European Gastroenterology (UEG) guidelines (44).

If pancreatic failure occurs, complete abstinence from alcohol and smoking is crucial for all patients to slow disease progression and alleviate pain. For those with malnutrition, malabsorption, or pancreatic exocrine insufficiency (PEI), pancreatic enzyme replacement therapy (PERT) is recommended to improve symptoms, nutritional status, and overall quality of life. A diet rich in protein and carbohydrates, while avoiding high-fat foods, is often advised to reduce malabsorption-related symptoms Additionally, regular blood glucose monitoring is essential for the early detection of hyperglycemia, which may develop as a complication of pancreatic insufficiency. If diabetes occurs, insulin therapy is typically required to manage blood sugar levels effectively (45, 46).

For patients presenting with jaundice and isolated bile duct narrowing, the first-line treatment involves ERCP with stent placement, combined with steroid therapy. The effectiveness of this approach is typically evaluated within 2–4 weeks. Surgical intervention is generally not recommended for IRC, except in cases where patients fail to respond to pharmacological and endoscopic treatments, particularly if they experience persistent severe abdominal pain (47). Surgery may also be necessary in cases of obstructive jaundice that do not resolve with endoscopic or medical therapies and in cases where pancreatic cancer cannot be definitively ruled out despite a thorough diagnostic evaluation (39). The challenges in achieving an accurate diagnosis are highlighted by The International Study Group of Pancreatic Surgery consensus statement, which noted that up to 13% of patients who underwent surgical resections for suspected malignancy were found to have benign pathology, among those - AIP in 30–43% cases (47).

### 4.1 Biliary tract

The bile ducts are the second most commonly affected site among IgG4-related disease. Guidelines recommend the term IgG4-related cholangitis (IRC) to emphasize its steroid responsiveness and the need for the differentiation from primary sclerosing cholangitis (PSC), bile duct carcinoma, or cholangiocarcinoma (39). IRC may occur independently or as an extra-pancreatic feature of IgG4-related AIP (48) and is seen in approximately 60%–80% of individuals with AIP (49–51). Clinically, IRC is linked to advanced age, with a higher prevalence in males. IRC symptoms include: obstructive jaundice, weight loss, and abdominal pain (52). Disease is often associated with diabetes mellitus. The biochemical features of IRC include elevated serum markers of cholestasis, such as alkaline phosphatase, gamma-glutamyl transferase, and conjugated bilirubin. The “tumor marker” CA19-9 can be significantly elevated in IRC and decreases quickly with steroid treatment (53). In the early stages, US findings are usually normal. Later, it may show circumferential bile duct wall thickening (intrahepatic, extrahepatic, or both) and intrahepatic bile duct dilatation. A characteristic feature is that the bile duct lumen remains visible despite wall thickening. However, US has low sensitivity for detecting the disease and cannot differentiate IRC from PSC or cholangiocarcinoma. IDUS (Intraductal Ultrasound) - an ultrasound probe inserted into the bile duct, shows the extent of bile duct wall thickening, with diffuse, symmetric, homogeneous thickening and smooth margins (54). CT is not the first-choice imaging for biliary diseases but may show bile duct wall thickening and mild dilatation of proximal ducts in cases of suspected AIP. Key findings include circumferential symmetric wall thickening, particularly in the extrahepatic bile ducts, with a visible lumen. Funnel-shaped dilatation of ducts proximal to strictures is also noted. MRI is the preferred imaging modality for IRC and AIP. Findings include bile duct wall thickening, single or multifocal involvement, smooth margins, long segment strictures, and visible lumens in thickened segments. Delayed homogeneous contrast enhancement and mass-like thickening (pseudotumor) in the hilar ducts may be seen. Gallbladder involvement (in about 51% of patients) appears as diffuse wall thickening and enhancement. ERCP was once commonly used for diagnosing ISC but now has limited utility due to MRI’s ability to provide detailed images of the biliary system on one side and the procedure invasiveness on the other. ERCP is primarily used when intervention (e.g., stent placement) is required. Its sensitivity in diagnosing ISC is 45%, with a specificity of 88% (55, 56).

Differentiation of IRC from cholangiocarcinoma is essential since the latter can also present with jaundice, bile duct obstruction, and mass-like lesions. Ct scans in cholangiocarcinoma often reveal an irregular mass or bile duct stricture, frequently accompanied by localized biliary dilatation. Tumor markers such as CA 19-9 are commonly elevated in cholangiocarcinoma, though these markers lack specificity and may occasionally be elevated in IRC (57). Unlike IRC, cholangiocarcinoma frequently demonstrates aggressive features on CT, such as vascular invasion, metastatic spread, and irregular or nodular bile duct thickening. Additionally, IRC typically resolves or shows significant improvement with steroid treatment, while cholangiocarcinoma, being a malignancy, is not responsive to steroid therapy. Additionally, while cholangiocarcinoma progresses despite corticosteroid therapy, IRC typically resolves or shows significant improvement under steroid treatment (58). Unlike AIP, serum IgG4 levels are more reliable indicators in IRC, being elevated in approximately 75% of cases. However, it is important to note that elevated serum IgG4 levels can also be observed in 3.2% of patients with cholangiocarcinoma and 15% of patients with PSC (19, 59).

Involvement of both, the pancreas and biliary system, together with elevated serum IgG4 levels strongly supports a diagnosis of IRC. However, in cases of isolated bile duct involvement, excluding malignancy remains the challenge. First, the incidence of biliary cancer is much higher than that of IRC, so the first is considered to begin with. Secondly, the sufficient material in biliary tract biopsy is technically difficult and seldom efficient. The absence of standardized diagnostic criteria for isolated IRC further contributes to the disease under-recognition. Therefore, the most important is the careful clinical evaluation of the whole picture and considering IgG4-RD, when necessary (60, 61).

IRC diagnostic criteria include lymphocyte and plasma cell infiltration with fibrosis, ratio of IgG4-positive plasma cells/IgG-positive cells > 40% and the number of IgG4-positive plasma cells > 10 per high powered field and typical tissue fibrosis, particularly storiform fibrosis, or obliterative phlebitis. In cases of biliary obstruction, high risk of cholangiocarcinoma, or failure of pharmacological treatment, surgical resection may be performed, allowing for the full spectrum of these features to be visualized. Nevertheless, the diagnostic histopathology after surgery is not practical. EUS can provide detailed imaging of the biliary system and pancreas, which may suggest disease involvement in the bile ducts, but does not directly confirm IRC. Key features seen on EUS include symmetric, diffuse bile duct wall thickening, narrowing or irregularities in the bile ducts indicative of inflammation or fibrosis associated with IRC, as well as pancreatic enlargement or masses that could suggest autoimmune pancreatitis (62). On the other hand, EUS-guided fine-needle biopsy (FNB), which is useful for obtaining samples from bile ducts in areas with focal lesions such as thickened bile duct walls or inflammatory changes, often presents diagnostic challenges, even for experienced pathologists (63). In addition, it was indicated that approximately 43% of bile duct carcinomas exhibit more than 10 IgG4+ plasma cells, a threshold commonly used to diagnose IRC (64).

Another clinical challenge is the differentiation of IRC from PSC. Mendes et al. reported that 9% of their PSC patients showed serum IgG4 > 140 mg/dL and in ampullary biopsies. MRI may help differentiate IRC from PSC, especially with bile duct wall thickness greater than 2.5 mm, continuous changes, gallbladder involvement, and absence of hepatic parenchymal involvement- characteristics that are more typical of IRC (65). MR cholangiography in PSC typically reveals the characteristic “beaded” appearance of the bile ducts, caused by alternating multifocal strictures and dilations. In contrast, IRC often presents long, smooth, segmental strictures accompanied by proximal biliary dilatation. While PSC demonstrates biliary fibrosis without significant IgG4-positive plasma cell infiltration, IRC biopsy findings typically include storiform fibrosis, obliterative phlebitis, and dense infiltration by IgG4-positive plasma cells. Finally, IRC responds effectively to steroid therapy, a key feature that distinguishes it from aforementioned conditions (66).

### 4.2 Liver

The involvement of the liver in IgG4-related disease is rather rare (67) and is accompanied with often nonspecific symptoms, including abdominal discomfort, fatigue, and jaundice. When it manifests as IgG4-associated autoimmune hepatitis, patients may experience symptoms similar to classic autoimmune hepatitis, such as malaise and elevated transaminases (68). Another possible liver manifestation is IgG4-associated inflammatory mass, which includes both a fibrohistiocytic type and a lymphoplasmacytic type (69). In some cases, liver lesions may be secondary to biliary obstruction from IRC or AIP or may arise from the direct spread of IRC into smaller bile ducts or portal tracts (70).

In later stages, liver involvement may appear like liver fibrosis or cirrhosis. IgG4-associated autoimmune hepatitis is often accompanied with hepatomegaly with irregular hypodense areas on CT, while MRI shows hypointensity on T2-weighted images and delayed contrast enhancement (22).

Histologically, IgG4-associated autoimmune hepatitis resembles classic autoimmune hepatitis but is distinguished by a dense lymphoplasmacytic infiltrate rich in IgG4-positive plasma cells, storiform fibrosis, and obliterative phlebitis (68).

Autoimmune hepatitis (AIH) should be differentiated from IgG4-RD due to overlapping features such as elevated liver enzymes. AIH is associated with autoantibodies such as anti-smooth muscle and anti-liver/kidney microsomal antibodies, which are generally absent in IgG4-RD. In addition, AIH primarily targets hepatocytes, causing hepatitis and periportal inflammation, whereas IgG4-RD typically involves bile ducts and portal areas. Histological examination reveals that AIH lacks the storiform fibrosis and dense infiltration of IgG4-positive plasma cells characteristic of IgG4-RD (71).

Primary biliary cholangitis (PBC), another chronic autoimmune hepatobiliary disease, shares features with IgG4-RD, including jaundice, fatigue, and cholestatic liver enzyme elevation. PBC is strongly associated with anti-mitochondrial antibodies (AMAs), a hallmark absent in IgG4-RD. Histologically, PBC is characterized by granulomatous inflammation and bile duct loss, contrasting with the storiform fibrosis and IgG4-positive plasma cell infiltration found in IgG4-RD. Furthermore, PBC typically follows a progressive course without significant response to corticosteroid therapy (71).

Gallbladder carcinoma may also resemble IgG4-related cholecystitis due to similar presentations of abdominal pain, jaundice, and gallbladder wall thickening on imaging. However, gallbladder carcinoma typically shows localized, irregular wall thickening with invasive features, while IgG4-related cholecystitis presents with diffuse thickening and lacks signs of malignancy. Histological findings in gallbladder carcinoma include malignant epithelial cells, whereas IgG4-RD reveals dense fibrosis and infiltration by IgG4-positive plasma cells. The good response to steroids in IgG4-RD, manifested by the resolution of symptoms and the halt or regression of changes observed in CT).

IgG4-related inflammatory masses should be differentiated from other mass-forming inflammatory conditions, which are characterized by a predominant histiocytic infiltrate and a lower density of IgG4+ plasma cells (72).

### 4.3 Salivary and lacrimal glands

IgG4-related disease can also affect the head and neck region, typically presenting with painless, persistent, or recurrent swelling of the salivary glands (sialadenitis) and/or tear glands (dacryoadenitis). These symptoms can result in noticeable changes in facial appearance (73, 74). In the past, this presentation was associated with Mikulicz’s disease but is now recognized as part of

IgG4-related dacryoadenitis and sialadenitis (75). It is crucial to differentiate this condition from Sjögren’s syndrome, which has overlapping clinical and radiological features but distinct histopathological characteristics.

Radiologically, salivary glands show homogeneous enhancement of the affected glands, which helps differentiate it from Sjögren’s syndrome, where a “salt and pepper” or “honeycomb” pattern is typically observed (Kamiński, 2020). In addition, the Küttner tumor—characterized by unilateral enlargement of the submandibular gland—is now classified as IgG4-related submandibular gland disease. This condition should primarily be differentiated from Sjögren’s syndrome. A clinical comparison between patients with Küttner tumor and those with Sjögren’s syndrome (SS) - related sialoadenitis revealed several key differences:

1. Patients with IgG4-RD experienced xerophthalmia (eye dryness due to reduced tear production), xerostomia (mouth dryness due to decreased saliva secretion), and arthralgia (joint pain without inflammation) less frequently than those with SS. However, they more often had coexisting autoimmune pancreatitis (AIP), interstitial nephritis, allergic rhinitis, and/or bronchial asthma.
2. Most IgG4-RD patients tested negative for anti-SS-A, anti-SS-B antibodies, rheumatoid factor (RF), and anti-nuclear antibodies (ANA), unlike SS patients.
3. Serum IgG4 and IgE levels were significantly higher in IgG4-RD than in SS.
4. Steroid therapy proved highly effective in IgG4-RD patients but had limited benefit in those with SS (76).

### 4.4 Other gastrointestinal manifestations

While IgG4-related disease frequently involves the liver and pancreas, it’s occurrence in oesophagus, stomach and intestines is rare and often regarded with skepticism. However, certain manifestations, such as gastric involvement, are well recognized. IgG4-RD can involve the gastrointestinal tract, presenting with ulcerations, polypoid lesions, submucosal masses, or wall thickening. Gastric involvement in IgG4-RD may appear as a mass-like lesion located in the fundus, body, antrum, or pylorus. On contrast-enhanced CT, the lesion typically presents as a homogeneously enhanced mass, with an enhanced linear structure representing the gastric mucosal layer, suggesting a submucosal location. MRI findings include a well-defined mass with low intensity on T1- and T2-weighted images and high intensity on diffusion-weighted images. A dynamic study may reveal a progressively enhancing pattern, with spared gastric mucosa visible in the arterial phase. Endoscopic and barium examinations often show a submucosal lesion compressing the gastric lumen, with the overlying mucosa appearing normal, though erosion or ulceration may not always be present. On EUS, the lesion is observed as a low-echoic mass located in the proper muscular or subserosal layer. In differential diagnosis, malignant lymphoma and gastrointestinal stromal tumor (GIST) should also be considered (77).

When IgG4-RD involves the gastrointestinal tract, it presents a range of nonspecific symptoms, such as abdominal pain, bloating, or discomfort, and may manifest as acute or chronic gastrointestinal issues (78, 79). Histological findings in biopsy specimens taken from the small intestinal wall or mucosal biopsies from areas of suspected involvement in the colon may include IgG4-positive cells exceeding the threshold of 50/HPF and IgG4/IgG ratios above 40% (80). When the appendix is involved, patients may experience symptoms resembling appendicitis, such as right lower abdominal pain, but typically without fever or leukocytosis (81, 82). Radiological findings observed through CT and MRI, can include thickened intestinal walls and submucosal masses. The stomach may show wall thickening or submucosal involvement, while the appendix can appear as a mass-like enlargement with thickened walls, periappendiceal or perimesenteric infiltration, and fat stranding. These features, along with the slow progression of symptoms, absence of fever, mildly elevated inflammatory markers, and possible involvement of other organs on CT, should raise suspicion for IgG4-RD and prompt the request for immunohistochemical staining in histopathological examination. These findings are often nonspecific and may mimic appendicitis or appendiceal tumors. It should also be emphasized that IgG4-RD affecting the appendix is very rare, and the available literature in medical databases consists only of individual case reports (77, 81–83).

Small-bowel involvement has also been documented, with some cases demonstrating disease affecting the serosal surface of the gastrointestinal tract. For instance, IgG4-RD case report has been reported as necrotizing mesenteric arteritis and a solitary jejunal ulcer (84). Many reported cases describe patients undergoing surgery for small bowel masses without prior testing for serum IgG4 levels. The most performed examination in these cases, which visualized these masses, is CT (85, 86). In the context of IgG4-RD affecting the digestive system, often reveal tumor-like proliferation which leads clinicians to perform a biopsy of the affected lesion. While a histopathological examination alone may not immediately confirm IgG4-RD, the pathologist may observe fibrosis, often in a storiform pattern, as well as vessel proliferation and obliterative phlebitis. These features raise suspicion and prompt the need for immunohistochemical testing, which can reveal lymphocytic infiltration and IgG4-positive plasma cells - this is crucial, as IgG4-positive plasma cell infiltration, with a typical IgG4/IgG ratio often exceeding 40%, is a hallmark of the disease and is essential for confirming the diagnosis of IgG4-RD (87). Early implementation of pharmacological treatment typical for IgG4-RD might have been effective in such cases, potentially avoiding unnecessary surgeries and their associated risks.

Elevated IgG4 levels in the blood are often observed in patients with eosinophilic esophagitis (EoE), which supports the fact that this condition must be considered during differential diagnosis in the context of gastrointestinal involvement in IgG4-related disease. EoE is a separate disease entity with a distinct pathogenic mechanism. It is a chronic inflammatory condition of the esophagus with an immunologic and allergic background, characterized by eosinophilic infiltration in the esophageal mucosa (88). EoE is associated with hypersensitivity reactions to food and airborne allergens. Symptoms such as dysphagia or food impaction have been reported (89, 90). Although nonspecific, an increased IgG4 levels were also seen in unrelated conditions like inflammatory bowel diseases (89).

### 4.5 Other clinical manifestations

Frequency of other organs involvement in IgG4-related disease are shown in **Table 1**. The PET-CT scan can be helpful in identifying other disease localizations (19). 18F-Fluorodeoxyglucose (18F-FDG) positron emission tomography/computed tomography (PET/CT) has proven valuable in assessing organ involvement, guiding biopsy, and monitoring treatment response in IgG4-RD. A key advantage of PET/CT is its high sensitivity and ability to evaluate multi-organ involvement in a single examination, surpassing conventional imaging methods such as standard computed tomography. However, its low specificity necessitates a thorough understanding of IgG4-RD imaging patterns to avoid misdiagnosis (91). A cohort study of 35 patients with IgG4-RD demonstrated that 97.1% had multi-organ involvement, with PET/CT detecting more affected organs than physical examination, ultrasound, or standard CT in 71.4% of cases. Specific imaging features included diffusely elevated uptake in the pancreas and salivary glands, patchy retroperitoneal and vascular lesions, and patterns distinguishable from metastases. PET/CT findings aided biopsy-site selection and interventional procedures such as ureteral recanalization (92).

**Table 1:** Frequency of organ involvement in IgG4-related disease (2, 14–16).

Organ	Frequency (%)	Comment
Pancreas	20–70%	Most frequently affected organs; symptoms include jaundice, abdominal pain, and diabetes.
Salivary Glands	20–30% in Europe; 60–80% in Asia	Often involved, especially submandibular glands, leading to land enlargement.
Orbital and Lacrimal Glands	10–50%	Painless enlargements, often associated with salivary gland involvement.
Lungs	10–30%	Can cause inflammatory pseudotumors, localized or diffuse interstitial pneumonia, and pleuritis.
Retroperitoneum	10–27%	Associated with retroperitoneal fibrosis (RPF), which may lead to obstructive uropathy.
Renal	7–24%	IgG4-related tubulointerstitial nephritis (TIN) is the predominant manifestation.
Vascular	10–20%	Affects large vessels such as the aorta; risks include aneurysm formation.
Mediastinum	5-15%	Rare fibrosing mediastinitis can compress vital mediastinal structures.
Thyroid	7-12%	May present as fibrosing thyroiditis, associated with gG4-RD.

IgG4-disease may involve the lacrimal glands, as well as the sclera, nasolacrimal duct, trigeminal nerve, extraocular muscles, orbital soft tissues, and even the optic nerve (93).

Renal involvement most commonly presents as tubulointerstitial nephritis (TIN), although membranous glomerulonephritis (MGN) and pyelitis may also occur (94). Obstructive nephropathy can develop due to IgG4-related retroperitoneal fibrosis or direct involvement of the ureters (95). Retroperitoneal fibrosis primarily affects tissues surrounding the abdominal aorta and ureters, potentially leading to complications such as obstructive uropathy, renal artery and vein stenosis, kidney atrophy (96). IgG4-related periaortitis often targets the outer layer of the aorta, typically in the abdominal segment (97). Periarteritis can extend to other large or medium-sized arteries, such as the iliac, renal, splenic, and mesenteric arteries (63). In the cardiovascular system, IgG4-RD may manifest as pericarditis or involvement of the aortic valve (98, 99). In some cases, IgG4-RD causes regional or generalized lymphadenopathy (100). Thyroid involvement is suspected in conditions such as Riedel’s thyroiditis and the fibrosing variant of Hashimoto’s thyroiditis (73, 101).

### 4.6 Cancer risk

IgG4-related disease (IgG4-RD) has been increasingly recognized as a condition associated with an elevated risk of certain malignancies. A meta-analysis incorporating ten studies investigated the relationship between IgG4-RD and cancer risk. The results demonstrated that patients with IgG4-RD exhibit a significantly increased risk of developing cancer compared to the general population. The standardized incidence ratio (SIR) for overall cancer in IgG4-RD patients was 2.57 (95% CI: 1.72–3.84). Notably, the risks for PDAC cancer and lymphoma were markedly elevated, with SIRs of 4.07 (95% CI: 1.04–15.92) and 69.17 (95% CI: 3.91–1223.04), respectively (102). In a study performed by Keller-Sarmiento, thirty-seven out of 210 patients affected by IgG4-RD, representing 18%, developed cancer either prior to or following their diagnosis of IgG4-RD. The most reported cancers were prostate cancer, melanoma, and gastric cancer, accounting for 15%. Interestingly, in males, the SIR was 2.78 times higher (p = 0.005), whereas in females, it was 1.15 (103).

In a cohort study by Zachary S. Wallace et al. conducted in 2016, 20 out of 125 patients diagnosed with IgG4-RD had a history of malignancy, representing 16%. The average age at malignancy diagnosis was 51.0 ± 18.0 years. Among these 20 patients, 18 had active disease, and 14 had elevated serum IgG4 levels (>135 mg/dl) at the time of their initial evaluation. The malignancy was diagnosed an average of 8.8 years before the IgG4-RD diagnosis. The most common malignancies were prostate cancer (7 cases) and lymphoma (4 cases). Among the lymphoma cases, there was 1 case of Burkitt’s lymphoma, 2 cases of non-Hodgkin’s lymphoma, and 1 case of diffuse large B-cell lymphoma. Other malignancies included breast, lung, and colorectal cancer (2 cases each), as well as leukemia and cervical cancer (1 case each) (104).

Patients with AIP itself are at a significantly increased risk of developing various types of cancers. The study by Shiokawa et al. conducted a multicenter retrospective cohort analysis in 108 AIP patients among which, 18 cancers were detected in 15 individuals (13.9%). The cancer risk in AIP patients was estimated to be about 2.7 times higher than in the general population. This risk was particularly elevated during the first year after the AIP diagnosis, being approximately six times higher, and then decreased in the following years to about 1.5 times higher (105).

During an international symposium on IgG4-related disease held in Boston in 2011, pathologists were presented with guidelines for diagnosing IgG4-RD. The main histopathological features highlighted include: 1. Dense lymphoplasmacytic infiltrate, 2. Fibrosis, often arranged in a storiform pattern, at least focally, 3. Obliterative phlebitis. Additionally, other histopathological findings associated with IgG4-RD include: 1. Phlebitis without complete lumen obliteration and 2. Increased numbers of eosinophils. However, it is important to note that the latter two features, when observed alone, are neither sensitive nor specific enough to confirm the diagnosis of IgG4-RD (17). If histological analysis, performed after biopsy and guided by the Boston criteria, raises any uncertainty, surgical resection should be considered. The detection of IgG4 antibodies in malignant tumors has led to speculation that IgG4-RD could represent a paraneoplastic sign. Akahoshi et al. described cases where IgG4-RD was diagnosed simultaneously with the onset or recurrence of cancer or within one year of cancer detection. The treatment of cancers, including tumor resection or chemotherapy, led to regression of IgG4-RD symptoms. Additionally, the authors reference a report examining the association between AIP and cancer, suggesting that AIP may, in some cases, developes as a paraneoplastic syndrome. This is supported by observations that no relapses of AIP occurred following successful cancer treatment. These findings underscore the importance of consideration in IgG-4 disease (106).

## 5 IgG4 diseases treatment

Induction of remission

Steroids (GS) are recommended for managing IgG4-RD as the primary treatment option to induce remission in patients exhibiting symptoms or active disease (107). The main contraindications for the use of GCs in IgG4-RD are typical and include active infections, severe diabetes, peptic ulcers or gastrointestinal bleeding, psychiatric disorders, uncontrolled hypertension or osteoporosis (108, 109). Treatment typically begins with a dose of prednisone or prednisolone at 0.6 mg/kg/day, which corresponds usually with a dose of 30 to 40 mg/day (110). In clinical practice, the starting dose of GCs can vary significantly, with some studies indicating the use of higher doses for patients experiencing severe complications, such as those affecting the pancreas, lungs or kidneys. After 2 to 4 weeks, the dosage can be gradually reduced based on the patient’s response and the disease’s severity. GCs therapy can induce remission in 82–100% of patients, particularly depending on the extent of organ involvement. Remission rates tend to be higher in patients with organ-limited disease compared to those with more systemic involvement. Following successful induction, some patients may benefit from ongoing maintenance therapy, which may incorporate steroid-sparing drugs (4, 110–112).

### 5.1 Steroids maintenance therapy

GCs maintenance therapy is recognized as an effective strategy for preventing relapses in IgG4-RD, particularly in cases of AIP (at least 5 mg of oral prednisolone daily). Maintenance therapy is particularly advised for patients exhibiting certain risk factors, including:

1. Discontinuing GCs after a short time
2. High IgG4 levels at the time of diagnosis/high IgG4 levels after GCs therapy,
3. Diffuse pancreatic enlargement,
4. Delayed radiological response,
5. Presence of more than two extra-pancreatic lesions,
6. Concurrent proximal IgG4-sclerosing cholangitis prior to treatment.

It is important to note that the practice of long-term GCs maintenance is more prevalent in Japan and South Korea, while it remains less common in European clinical settings (25, 41, 113, 114). For patients without the risk factors mentioned above who have achieved remission, GCs doses should be gradually tapered up to the complete discontinuation (115).

### 5.2 Assessment of treatment response

The evaluation of treatment response in IgG4-RD requires a comprehensive and multi-dimensional approach, integrating clinical, laboratory, radiological, and histopathological assessments to determine both the effectiveness of therapeutic interventions and the progression or remission of the disease. IgG4-RD is a disease that responds well to treatment, with patients often showing improvement within days to weeks of starting effective therapy. However, it is important to acknowledge that in certain cases, organ damage may have already occurred by the time of diagnosis, and initiating treatment may not fully reverse the baseline damage. The pancreas, in particular AIP, serves as a key example for this phenomenon (116, 117).

These clinical improvements are typically correlated with the decrease in disease activity, which can be monitored through standardized scoring systems such as the IgG4-RD Responder Index (IgG4-RD RI). This index evaluates the response by assessing changes in organ-specific manifestations and systemic features, with a reduction in the score signifying a positive treatment outcome. In addition to its clinical utility, the IgG4-RD RI has been validated in several studies and has been incorporated into international consensus guidelines for the management of IgG4-RD. Its use helps standardize the assessment of treatment response and enhances comparability across different clinical settings and research studies. The calculation of the RI involves assessing disease activity on an organ-by-organ basis, with individual organ scores contributing to a total score. Investigators evaluate disease activity and damage across 24 predefined organs/sites. Additionally, constitutional symptoms—such as weight loss, fever, and fatigue—are considered a 25th domain of disease activity. Disease activity in each organ or site is rated on a scale from 0 to 4, with the following classifications:

0 = Unaffected or resolved
1 = Improved but persistent
2 = New or recurrence (while off of treatment) or unchanged
3 = Worse or new (despite treatment) (118)

In addition to clinical evaluation, laboratory markers such as serum IgG4 levels are often used to gauge disease activity. However, it is important to recognize that elevated serum IgG4 levels do not always directly correlate with the severity of IgG4-RD, and their normalization does not always mean the complete disease resolution. Therefore, serum IgG4 levels should be interpreted with caution and considered alongside other clinical and diagnostic findings (119). Imaging studies, including CT, MRI and US, are often indicative of a favorable treatment response and are commonly used to track disease progression or remission. CT may show a decrease in organ size during follow-up, indicating a positive treatment response, while MRI can provide additional insights by detecting tissue composition changes, such as a reduction in T2-weighted signal intensity, which suggests diminished inflammation and fibrosis (119). Finally, the ability to taper the dose of steroids without triggering a relapse of disease symptoms is an important clinical marker of treatment success. Given that steroids are the first-line therapy for IgG4-RD, a successful dose reduction is considered a key indicator of sustained disease control and treatment efficacy. Overall, the assessment of treatment response in IgG4-RD necessitates a holistic and integrated approach, incorporating clinical, laboratory, radiological, and histopathological data to provide a comprehensive understanding of the disease’s progression and the patient’s response to therapeutic interventions (120).

### 5.3 Second-line therapy

**Figure 5:**
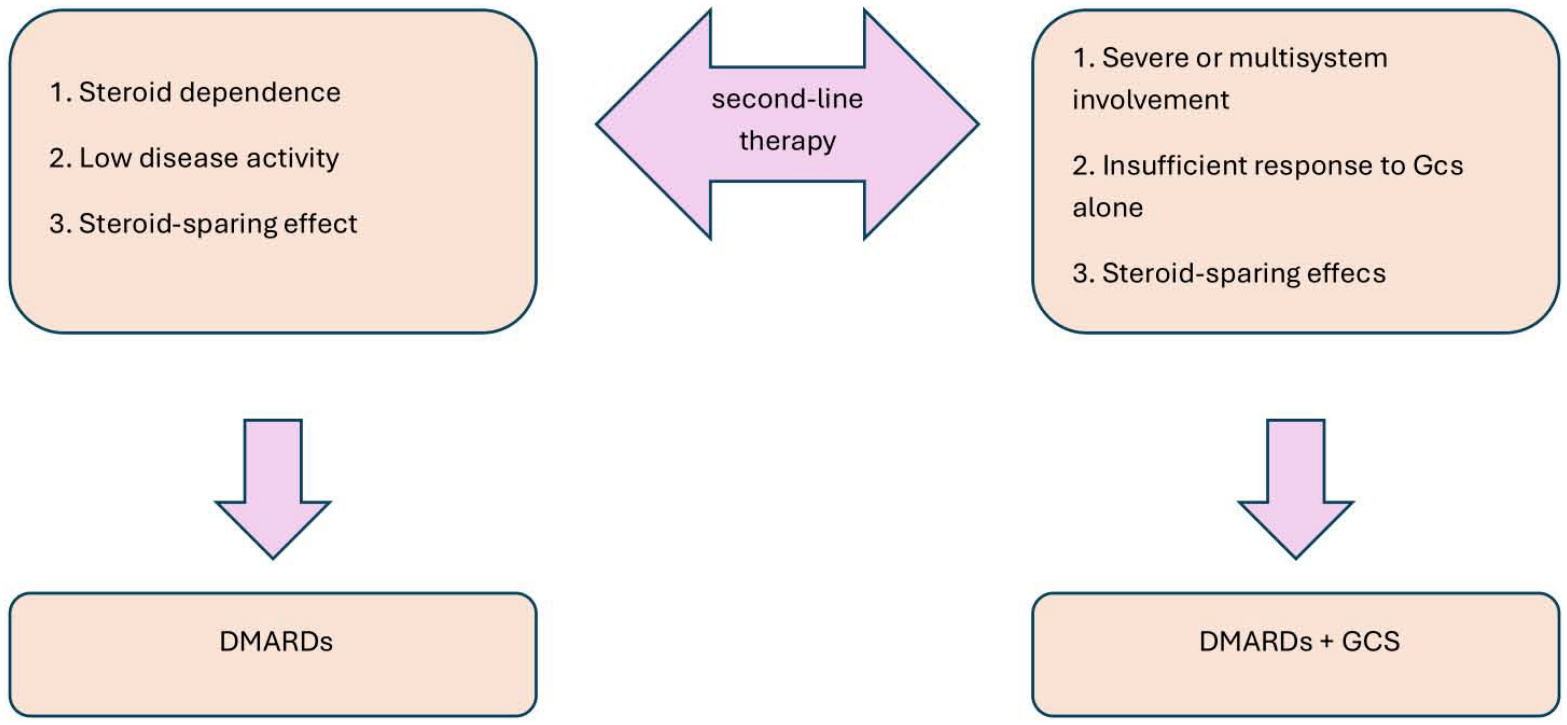
Strategy of second-line treatment in IgG4-RD (112, 121).

When there is a poor response to steroids or if there are steroid-dependent relapses, disease-modifying antirheumatic drugs (DMARDs) are used. The decision to use DMARDs in combination with steroids or as monotherapy depends on the severity of the disease and the patient’s response to initial treatment, as shown in **Figure 5**. DMARDs provide a steroid-sparing effect, whether used alone or in combination with GCs (112, 121).

DMARDs function by modulating immune activity to reduce inflammation and tissue damage, which restrains the IgG4-RD progression. Azathioprine (AZA) and mycophenolate mofetil (MMF) inhibits purine synthesis, thereby reducing lymphocyte proliferation, methotrexate (MTX) inhibits folate pathways in actively dividing cells and cyclophosphamide (CYC) alkylates DNA in proliferating cells (122, 123). They are often used as maintenance therapy following GC induction, particularly in cases where recurrent relapses occur. In the study conducted by Shangzu sixty-nine patients newly diagnosed with IgG4-RD were randomly assigned to two groups: Group I (35 patients) received GCs monotherapy at a dose of 0.6-0.8 mg/kg/day, gradually tapered, while Group II (34 patients) received a combination of GCs and MMF at 1-1.5 g/day. Results indicated that while both groups showed similar efficacy at the 1-month mark, the complete response rate in Group II was significantly higher at next follow-up points - the criterion for improvement in the overall follow-up was the sustained remission rate. The cumulative relapse rate over one year was also higher in Group I compared to Group II (40.00% vs. 20.59%), suggesting that addition of MMF may reduce the risk of relapse. Additionally, the remission rate was lower in Group I (51.42% vs. 76.47%) (124). MTX also presents a promising approach for maintaining remission induced by GCs what was shown in the 10 patients group with IgG4-RD. MTX was introduced after the initiation of GCs therapy, with the dosage escalated to 20 mg per week. At the time of MTX introduction, the mean daily dosage of prednisone was 20.75 mg (range 10-50 mg). After six months of MTX therapy, three patients who had achieved complete remission (CR) were able to discontinue GCs treatment. By twelve months, five patients remained in CR without prednisone, while the other five were in partial remission (PR) on a maintenance dose of prednisone at 5 mg/day. Interestingly, no relapses were observed under MTX treatment, as assessed using the Responder Index (evaluated by examining 24 standard organs/sites and applying a scoring system where each affected organ/site was graded on a scale from 0 to 4) (4, 111).

However, compared to other second-line therapeutics CYC possibly demonstrates higher efficacy, especially in severe cases with multi-organ involvement. In the case report by Zhang et al., the patient was treated with GCs (methylprednisolone at 250 mg/day for 3 days, followed by 40 mg/day) in conjunction with CYC (administered intravenously at a dose of 0.8 g per month). This therapeutic regimen resulted in the normalization of serum IgG4 levels, as well as significant improvement in the patient’s renal dysfunction and pulmonary lesions (125, 126). A retrospective cohort study conducted by Luo et al. included 155 IgG4-RD patients who received glucocorticoids (GCs) in combination with either CYC or MMF. Group I, patients were initially treated with an oral dose of CYC at a mean of 54.75 mg/day (ranging from 50 to 100 mg/day), which was maintained for 3 months before being reduced to 50 mg per day or every other day, in line with established treatment regimens. The mean cumulative dose of CYC over 12 months was 11.30 grams. In Group II, patients received an initial dose of MMF at a mean of 1,060 mg/day (ranging from 1,000 to 1,500 mg/day), which was sustained for 6 months before being reduced to 500 - 1,000 mg/day for the following 6 months, according to standard protocols. At the final follow-up, the overall response rate was 98.15% in Group I and 96.3% in Group II. However, within 12 months, the cumulative relapse rate was significantly higher in Group II compared to Group I (14.8% vs. 3.7%, P = 0.046), showing that CYC, when combined with GCs, resulted in better relapse prevention compared to MMF (127). AZA and MTX can lead to hepatotoxicity and bone marrow suppression, while also presenting risks for infection due to immunosuppression. MMF is rather well tolerated but may cause gastrointestinal upset and hematologic side effects. CYC is generally reserved for more aggressive disease manifestations due to its strong immunosuppressive effect and associated risks for serious side effects - significant cytopenia and infection risk (121, 128).

### 5.4 Third-line therapy:

In cases where conventional treatments fail, including steroid-refractory or steroid-dependent disease, or when relapses occur despite prior treatment - within 12 months of stopping Gcs, or when administration of GCs is contraindicated, when biochemical and imaging findings suggest the potential for life-threatening complications, such as organ failure (e.g. TIN or coronary involvement), the American College of Rheumatology guidelines, along with other consensus statements, advocate for the use of rituximab (RTX) or alternative biologic therapies. RTX is a monoclonal antibody that specifically targets the Cluster of Differentiation 20 (CD20) protein present on the surface of B lymphocytes. By binding to CD20, RTX induces the depletion of B cells, which play a pivotal role in the pathogenesis of IgG4-RD (129).

RTX is recommended for patients who are resistant or intolerant to high-dose GCs, require remission maintenance, or have not responded to immunosuppressive therapies. The dosing regimen involves administering 375 mg/m² of body surface area weekly for four weeks, followed by infusions every 2–3 months, or two 1000 mg infusions 15 days apart every six months. Besides GCs, rituximab is the only medication proven to induce remission in IgG4-RD (39). RTX has proven to be highly effective in reducing disease relapses, minimizing the need for prolonged steroid use, and achieving remission in cases of IgG4-RD that threaten vital organs. In one study, 80% of patients showed significant improvement or reached remission following treatment with RTX (130). A meta-analysis of 18 studies involving 374 patients revealed the following findings for RTX induction therapy. The pooled response rate was exceptionally high at 97.3%, while complete remission was achieved in 55.8% of patients. The overall relapse rate was 16.9%, and the pooled adverse event rate was 31.6%. Serious adverse events were rare, occurring in only 3.9% of cases (131). Studies indicate that RTX treatment can lead to long-lasting remission, with some individuals remaining in remission for years following a single course of therapy (132). RTX is generally well-tolerated, but it can lead to side effects, especially in patients with compromised immune systems. RTX can elevate the risk of infections, particularly opportunistic ones like Hepatitis B reactivation or Pneumocystis pneumonia. To mitigate these risks, regular screening and preventive measures are essential for specific patient groups. Additionally, RTX can cause depletion of blood cells, especially neutropenia or thrombocytopenia, though these effects are generally reversible. Hypogammaglobulinemia, which affects around 40-50% of patients, can sometimes persist long-term, heightening the risk of severe infections (133–136).

Although RTX has so far provided the most convincing evidence of efficacy in the treatment of third-line IgG4-RD, it is important to pay attention to the latest scientific reports regarding inebilizumab (INN), which could potentially change the current therapeutic paradigm.

INN is a humanized IgG1κ monoclonal antibody that targets CD19, a surface antigen expressed more broadly and at earlier stages of B-cell development compared to CD20. This allows it to achieve rapid, profound, and sustained depletion of a wider spectrum of B cells, including plasmablasts and some plasma cells—key drivers of disease activity in IgG4-RD. As such, INN may offer mechanistic and clinical advantages over CD20-targeted therapies.

The pivotal phase 3 MITIGATE trial confirmed the therapeutic potential of INN in IgG4-RD. Treatment significantly reduced the risk of adjudicated disease flares by 87% and the annualized flare rate by 86%, compared to placebo. Furthermore, 59% of patients receiving INN achieved flare-free, glucocorticoid-free complete remission at week 52, versus 22% in the placebo group. Notably, 90% of patients in the INN group discontinued GCs therapy entirely during the treatment period, underscoring its steroid-sparing effect.

While the short-term safety profile appears acceptable, a slightly increased rate of serious adverse events and infections was observed, highlighting the importance of ongoing safety monitoring. A 3-year open-label extension study is currently underway to provide further insight into long-term outcomes (137, 138).

### 5.5 Other targeted therapies:

Although several studies have explored the efficacy of alternative biologic agents in refractory IgG4-RD, the available evidence is predominantly derived from case reports or studies with limited patient cohorts. In a prospective study comparing tocilizumab (TCZ) - an IL-6 receptor inhibitor, and CYC in treating active IgG4-RD, TCZ showed improved clinical outcomes. After six months, 50% of the TCZ group achieved a complete response, compared to 20% in the CYC group, though the difference was not statistically significant. Notably, the TCZ group required significantly less GCs use. TCZ also had a comparable curative effect and better tolerance, suggesting it may be a more effective steroid-sparing option than CYC in managing IgG4-RD.

A study evaluating the use of abatacept (ABA) - a T-cell costimulation modulator (CTLA-4-Ig), in 10 patients with active IgG4-RD revealed promising but variable results. Over 24 weeks of weekly subcutaneous ABA (125 mg), 60% of patients responded by week 12, and 30% achieved complete remission by week 24. Half of the participants discontinued treatment due to flares or lack of response, and one adverse event (grade two thrombocytopenia) was reported.

Rigorous, large-scale clinical trials are needed to evaluate the effectiveness of these therapies and to establish their role as the potential second-line treatments for IgG4-RD.

Below is a summary of drugs, other than RTX, that may have off-label use in targeted therapy for IgG4-RD, presented in **Table 2**.

**Table 2:** A summary of drugs in targeted therapies potentially used for IgG4-RD (139–147).

Substance	Mechanism	Registration in other diseases
Tocilizumab (TCZ)	IL-6 inhibitor	Rheumatoid arthritis, juvenile idiopathic arthritis, giant cell arteritis
Abatacept (ABA)	T-cell costimulation modulator	Rheumatoid arthritis, juvenile idiopathic arthritis, psoriatic arthritis
Infliximab (IFX)	TNF-α inhibitor	Crohn’s disease, ulcerative colitis, rheumatoid arthritis, psoriasis
Ocrelizumab (OCR)	Anti-CD20 monoclonal antibody	Multiple sclerosis (relapsing-remitting and primary progressive forms)
Ustekinumab (UST)	IL-12 and IL-23 inhibitor	Psoriasis, psoriatic arthritis, Crohn’s disease, ulcerative colitis
Dupilumab (DUP)	IL-4 and IL-13 inhibitor	Atopic dermatitis, asthma, chronic rhinosinusitis with nasal polyps

### 5.6 When should surgical treatment be considered?

A. Rapidly progressing risk of organ failure:

The effects of GC treatment are achieved within days to several weeks, while the effects of RTX treatment take from 3 weeks (12, 148). In some cases, the dynamics of Gs response may be too slow to relieve symptoms quickly enough in the context of progressively increasing organ dysfunction. Conditions that may arise from advancing IgG4-RD include severe hydronephrosis and acute renal failure due to ureteral obstruction, significant biliary obstruction, or tracheal and esophageal compression. In these situations, rapid surgical intervention may be necessary to restrain the disease progression, prevent serious complications, and create a stable condition to initiate pharmacological therapy. It is important to note that surgical treatment of IgG4-RD can often result in disease regression. In a small study by Karim AF et al., patients who were initially treated with surgery showed improvement, with a reduced rate of recurrence after long-term follow-up (121, 149).

B. When organ involvement has become severe and irreversible:

and pharmacological treatment alone cannot reverse the damage, as in sclerosing mesenteritis. The surgery may be necessary to prevent ischemic necrosis of the bowel. If ischemic necrosis has already occurred, surgical intervention is required to remove the necrotic tissue (150–152).

## 6 How to prevent mismanagement in treatment?

**Figure 6:**
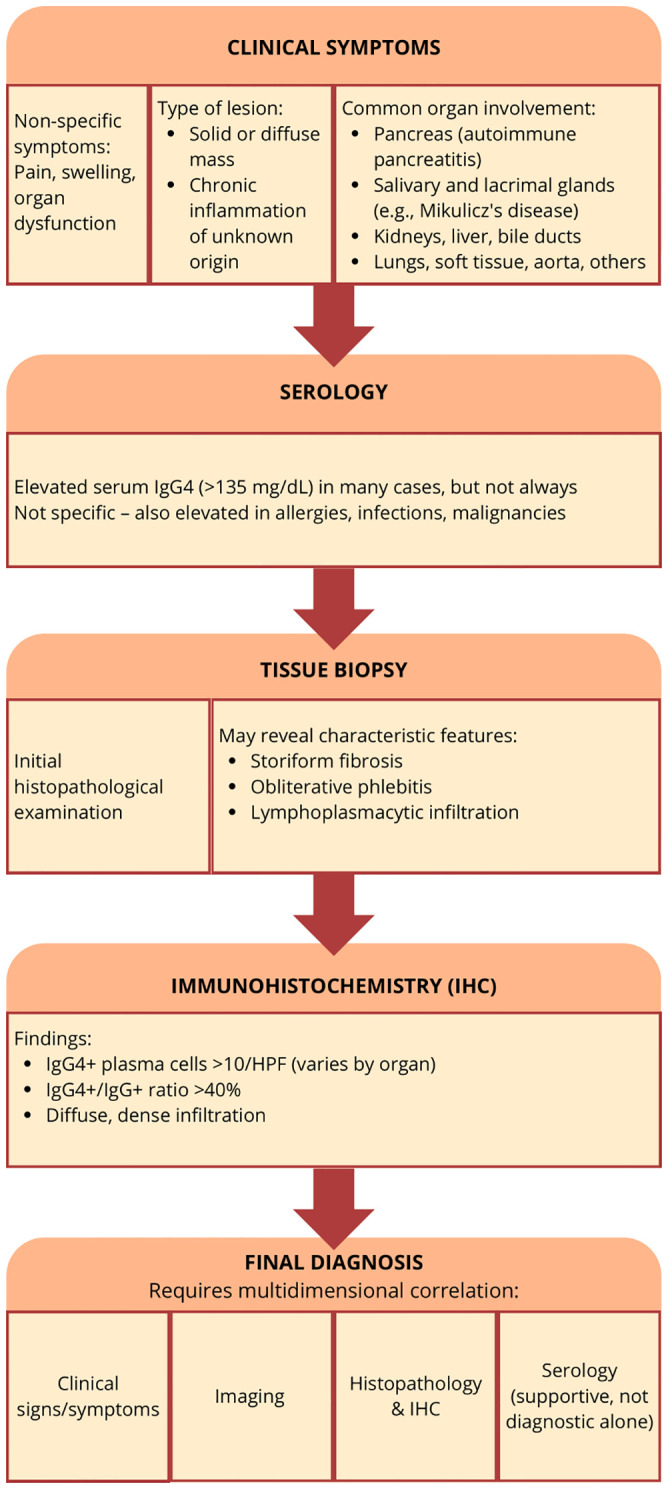
Summary of the therapeutic strategy in the management of IgG4-RD.

**Figure 6**. Summary of the therapeutic strategy in the management of IgG4-RD

To prevent mismanagement in IgG4-RD, it’s crucial to recognize that the disease can often be indolent and asymptomatic. Due to the limited sensitivity and specificity of diagnostic tests as well as not enough IgG4 disease awareness, unnecessary surgery is being introduced for patients who could benefit from systemic steroid therapy. Clinicians should pay particular attention to atypical symptoms that may accompany IgG4-RD. For instance, in cases of suspected pancreatitis with mild pain or in patients already diagnosed with IBD, it may be beneficial to measure serum IgG4 antibody levels or opt for the measurement of other markers like CRP, eosinophils, CA19-9 and bilirubin levels, depending on the affected organs (153).

The greater difficulty in diagnosing the disease in Europe is likely due to the higher prevalence of type 2 AIP, where serum IgG4 levels are typically normal, compared to Asia (34).

Biopsy with histopathological examination remains the gold standard for confirming IgG4-RD. The sensitivity and specificity of biopsy in diagnosing IgG4-RD depend on the histopathological features assessed by the pathologist. Masaki Y et al. emphasized the importance of calculating the IgG4/total IgG ratio. Their study demonstrated that a cut-off value of 40% achieved a sensitivity of 94% and specificity of 86%, making it one of the most sensitive tissue-based markers for IgG4-RD. However, certain histopathological features, such as lymphocyte and plasma cell infiltration with fibrosis, the IgG4/IgG ratio, and particularly storiform fibrosis or obliterative phlebitis, are not uniformly present across all organ manifestations of the disease. For instance, storiform fibrosis and obliterative phlebitis may be minor or entirely absent in organs such as the lacrimal glands, salivary glands, lymph nodes, lungs, or kidneys. Immunohistochemical staining is crucial for diagnosing IgG4-RD, as it allows for the identification of IgG4-positive plasma cell infiltration and fibrosis, as well as the storiform pattern within tissue samples. If the suspicion of the disease is not raised by the clinician and immunohistochemical staining of the samples is not ordered, the biopsy samples will lack the diagnostic value (17, 101, 154, 155).

IgG4-RD can affect multiple organs in 60%–90% of patients. Early recognition is crucial, as IgG4-RD can mimic other conditions, severely impact quality of life, and, when vital organs are involved, delayed diagnosis can be life-threatening - study reported a mortality rate of 10% (156). Remission maintenance strategies are inconsistent, and their long-term use poses challenges, particularly for elderly patients. The primary goal of treatment is to prevent fibrosis, as therapeutic options are limited once fibrosis becomes established. To achieve an accurate diagnosis, clinical features must be carefully correlated to imaging, histological architecture, and immunohistochemical staining (157). Comprehensive diagnostic criteria are essential for differentiating IgG4-RD from similar conditions, such as PDAC or biliary cancer. Biological therapies may provide new options for remission maintenance and for targeting fibrotic lesions thus early intervention and a personalized approach to treatment are essential to improving long-term outcomes (158).

A potential solution for improving diagnostic accuracy is to enhance collaboration between specialized centers, elaborate the disease registers and biobanks in high-referral centers. Such specialized teams can provide more precise diagnoses, especially when histological confirmation is not available (159). To establish effective treatment protocols, large-scale multicenter clinical trials are also necessary. The guidelines highlight the importance of coordinated efforts among different subspecialties involved in the diagnosis and management of the disease, such as radiologists and pathologists. Although pathology is considered the definitive method for diagnosing IgG4-related disease, it is not always available and is generally just one part of a more comprehensive and complex diagnostic strategy (39).

In the last 15 years IgG4-RD from unrecognizable disease became the one being considered and diagnosed all over the world. This is the object of interest of the clinicians of many specialties, which need to be aware of this pathology. IgG-RD is mimicking numerous more frequent and often threatening diseases, which causes many clinical decisions to be difficult. It may slowly develop with no or scarce symptoms causing serious organ damage. On the other hand, the great progress in the IgG-RD management and the disease, once recognized, may be now very successfully treated.
